# Automatic determination of malate dehydrogenase activity with a flow-injection multidetection system

**DOI:** 10.1155/S1463924690000062

**Published:** 1990

**Authors:** J. M. Fernández-Romero, M. D. Luque de Castro

**Affiliations:** Department of Analytical Chemistry Faculty of Sciences University of Córdoba Córdoba 14004 Spain

## Abstract

An automatic flow-injection method for the determination of malate dehydrogenase activity is proposed. The manifold used includes a selecting valve for closing the circuit along which the reacting plug is continuously circulated, and passed through the flow-cell of a conventional spectrophotometer, inserted into the closed circuit. A multipeak recording is obtained, each peak corresponding to one passage of the plug through the flow-cell. This recording allows the sensitivity to be modified, as required, using different types of measurements (for example, absorbance of the maxima or minima and absorbance differences between maxima or minima or their sums). The activity of the analyte can be determined in the range 0.02-2.00 U/l, with an r.s.d, of less than 0.90% at a sampling rate between 11 and 65 samples/h. The applicability of the method was checked by applying it to human serum samples. Analyte recoveries between 96 and 103% were obtained.


Journal of Automatic Chemistry, Vol. 12, No. 2 (March–April 1990), pp. 49–52

### Automatic determination of malate dehydrogenase activity with a flow-injection multidetection system

**J. M. Fernández-Romero and M. D. Luque de Castro**
*Department of Analytical Chemistry, Faculty of Sciences, University of Córdoba, 14004 Córdoba, Spain*

*An automatic flow-injection method for the determination of malate dehydrogenase activity is proposed. The manifold used includes a selecting valve for closing the circuit along which the reacting plug is continuously circulated, and passed through the flow-cell of a conventional spectrophotometer, inserted into the closed circuit. A multipeak recording is obtained, each peak corresponding to one passage of the plug through the flow-cell. This recording allows the sensitivity to be modified, as required, using different types of measurements (for example, absorbance of the maxima or minima and absorbance differences between maxima or minima or their sums). The activity of the analyte can be determined in the range 0·02–2·00 U/l, with an r.s.d. of less than 0·90% at a sampling rate between 11 and 65 samples/h. The applicability of the method was checked by applying it to human serum samples. Analyte recoveries between 96 and 103% were obtained.*

### Introduction

Flow injection analysis (FIA) has proved to be a useful tool for clinical analyses [1–3]. Although the FIA literature is rich with enzymatic methods, there are few reports of its use for the determination of enzyme activities [4] despite FIA's potential and adaptability to the different needs encountered in this field.

This paper reports a method for the determination of malate dehydrogenase (MDH) activity. The activity of this enzyme (L-malate: $NAD^+$ oxido-reductase EC 1.1.1.27) is increased as a result of such conditions as myocardial infarction [5], liver diseases [6] and post trauma [7]. The proposed method relies on the reduction of oxaloacetate to malate in the presence of the reduced form of the coenzyme:

$$\text{Oxaloacetate} + H^+ + \text{NADH} \underset{}{\overset{\text{MDH}}{\rightleftharpoons}} \text{Malate} + \text{NAD}^+$$

The reaction is monitored photometrically at 340 nm through the absorbance decrease caused by NADH consumption.

The FIA configuration used for this purpose allows multidetection with a single conventional detector in an open-closed design [8,9], which provides multipeak recordings in which each peak corresponds to one passage of the reacting plug through the single detection point within different proportions of reaction development. This allows kinetic measurements between peaks (whether consecutive or not) to be performed. In addition, the expansion of the sensitivity and determination ranges of the analyte can be readily obtained [10] by adding up several analytical signals, measuring signal maxima or minima.

Correspondence to: Dr M. D. Luque de Castro.

### Experimental

### *Reagents*

A 10 mM NADH (Sigma cat. no. N-8129, Sigma Chemical Company, St. Louis, Missouri, USA) solution in 30% ethylenglycol, a 50 mM oxaloacetate (Sigma cat. no. O-4126) solution in 0·1 M phosphate buffer of pH 7·0, a 1000 U $l^{-1}$ MDH solution (Sigma cat. no. M-2634) in 0·1 M phosphate buffer of pH 7·0 and a 0·1 M potassium dihydrogenphosphate buffer of pH 7·0 were used. From these, more diluted solutions were prepared by dilution with 0·1 M phosphate buffer of pH 7·0. All reagents used were of analytical grade.

### *Apparatus and instruments*

A Pye-Unicam (Pye Unicam Ltd., Cambridge, UK) SP-500 single-beam spectrophotometer equipped with a Hellma 178.12QS flow-cell was connected to a Radiometer (Radiometer, Copenhagen, Emdrupre, Denmark) recorder. A four-channel Gilson Minipuls-2 peristaltic pump with a rate selector, two Rheodyne (Rheodyne Inc., Cotati, California, USA) 5041 injection vales (one modified to act as a selecting valve), a Tecator (Tecator AB, Höganäs, Sweden) TM-III chemifold and a Selecta 382-S thermostat were used. A Leo PC system equipped with a DAS-8PGS interface (Metrabyte Co., Taunton, Massachusetts, USA) collected the absorbance/time data and the absorbance values at the maxima and minima.

### *Manifold and procedure*

The sample (serum diluted with phosphate buffer as required) is aspirated through $P_1$ into the loop of the injection valve (IV), which inserts it into a buffer-carrier stream that is sequentially merged with the substrate (oxaloacetate) and the reduced form of the coenzyme (NADH). Reactor $L_1$ allows for adequate sample-reagent mixing and hence the reaction to be started. In this step, the selecting valve (SV) keeps the circuit open. After the reacting plug has passed through SV, this is switched to close the circuit; the carrier stream is driven to waste and the reacting plug is circulated continuously along the

0142–0453/90 $3.00 © 1990 Taylor & Francis Ltd.

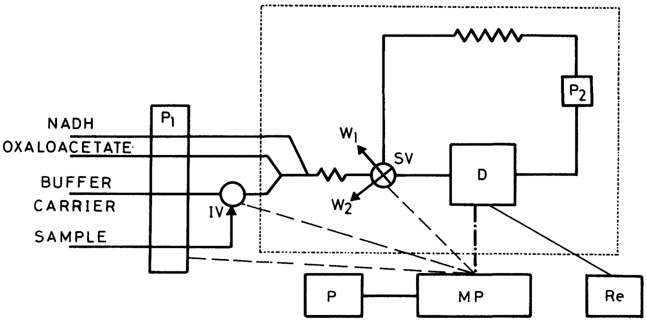

*Figure 1. Open-closed FIA manifold for the determination of MDH activity. $P_1$ and $P_2$ denote peristaltic pumps; IV and SV are injection and selecting valves, respectively; $W_1$ and $W_2$ are waste; D is the detector and MC, P and Re are the microcomputer, printer and recorder, respectively. (The boxed zone was thermostated.)*

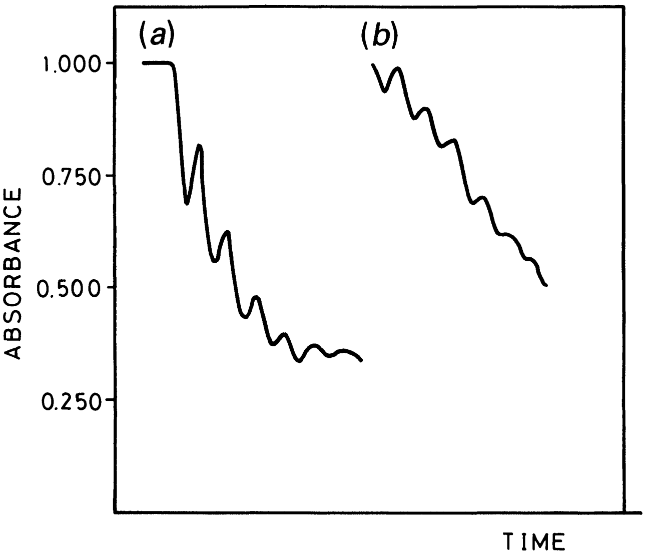

*Figure 2. Multipeak recordings for malate dehydrogenase activity. (a) 0·5 U/l, (b) 0·1 U/l.*

closed circuit with the aid of $P_2$. Each cycle provides a signal which is acquired by the computer or plotted through the recorder. The multiple data or multipeak recordings obtained are compared with the calibration curves obtained with standards treated as the samples stored in the computer or plotted through the recorder for subsequent calculation of the malate dehydrogenase activity by one of the different alternatives described below.

*Types of measurements*

Figure 2 shows the multipeak recording obtained with the open-closed manifold for the MDH/oxaloacetate/NADH system. The peaks of this recording correspond to absorbance decreases caused by the oxidation of NADH to $NAD^+$; the peak shape is dependent on the reaction rate, the ratio of injected volume to overall volume of the closed circuit and the flow-rate along it. The baseline level depends on the NADH concentration and is established at high absorbance.

The relationship between the analyte activity and the measured parameter can be established by measuring the absorbances at successive maxima peak ($A_1$, $A_2$, etc.) or minima ($A'_1$, $A'_2$, etc.), or from the sum of the absorbances of several maxima ($\sum_1^n A_i$) or minima ($\sum_1^n A'_i$) (i.e. fixed-time measurements). The absorbance increment over a given interval can also be used for this purpose; thus the absorbance increment can be measured between two maxima $\bar{v} = \sum_1^n (A_n - A_{n-1})/(t_n - t_{n-1})$, or two minima ($\bar{v}' = \sum_1^n (A_n - A_{n-1})/(t_n - t_{n-1})$) (i.e. reaction-rate kinetic methods). The information obtained from each standard used to construct the calibration graph can be treated in different w.ays. The plot obtained from the first peak can be regarded as the 'normal' calibration graph by analogy with normal flow-injection systems, which provide a single peak per injected sample. A fan-shaped set of calibration graphs with steeper slopes can be obtained if the peak maxima where the reaction has proceed to a greater extent or the sums of peak signals obtained at different times; the gentlest slopes are achieved by using the minima of the recordings. These alternatives provide a way of selecting the most suitable sensitivity compared with the normal method. The types of measurements used are summarized in table 1.

Some of the physico-chemical parameters that can be calculated are the apparent rate constant of the reaction involved and the conditional Michaelis constant, $K_m$. The latter can be calculated from the data obtained at different concentrations of substrate (oxaloacetate) by means of a Lineweaver–Burke plot.

*Results and discussion*

The influence of the variables affecting the system was studied by the univariate method. These variables can be classified into physical, chemical and FIA.

As the temperature is usually a key variable in enzymatic reactions, it was studied first by immersing all the reactors shown in the boxed zone in figure 1 into a water-thermostated bath and recirculating water at the same temperature as the bath through the thermostated flow-cell compartment. The bath temperature was varied from 20 to 50 °C in 5 °C intervals. The temperature of the reactant plug never equalled that of the bath because the residence time was not long enough. The peak height increased with the temperature up to 40 °C, above which the signals kept constant or even decreased owing to the denaturation of the analyte. A temperature of 40 °C was chosen for further experiments.

The flow-rate provided by $P_1$ was not an influencial variable as it only affected the first peak in the recording. On the other hand, the flow-rate of $P_2$ conditioned the number of peaks obtained before homogenization of the overall volume in the closed circuit as well as reaction development between two consecutive peaks; 1·64 ml/min was chosen as a compromise.

An appropriate choice of the injected volume was essential for selecting the best circuit length and hence the

*Table 1. Types of measurements and methods from the multipeak recording.*

| Type | Method | Measured parameter | Calculation of the parameter |
|---|---|---|---|
| | Normal | Absorbance of the maxima | $A_1, A_2, A_3, \ldots$ |
| At fixed-time | Dilution | Absorbance of the minima | $A'_1, A'_2, A'_3, \ldots$ |
| | Concentration | Sum of absorbances of different maxima | $\sum_1^n A_i$ |
| | | Sum of absorbances of different minima | $\sum_1^n A'_i$ |
| | Normal | Difference of signal between maxima | $\bar{v}_i = \frac{(A_i - A_{i-1})}{(t_i - t_{i-1})}$ |
| Reaction-rate | Dilution | Difference of signal between minima | $\bar{v}'_i = \frac{(A'_i - A'_{i-1})}{(t'_i - t'_{i-1})}$ |
| | Concentration | Sum of the above signals | $\sum_1^n \bar{v}_i, \sum_1^n \bar{v}'_i$ |

optimum sample volume/overall volume ratio. The joint study of both variables yielded 100 μl and 100 cm as optimum injected volume and $L_2$ length, respectively (the inner diameter of $L_2$ was 0·5 mm). The length of $L_1$ was also studied in order to obtain the best $L_1$ value whenever this peak was used for calculation of the analyte concentration (its optimum value was 150 cm).

The selecting valve was switched after a preset time from injection to ensure the entire reacting plug was located in the circuit when it was closed. This was accomplished by closing the circuit 70 s after injection.

A 0·1 M phosphate buffer concentration was chosen as the most appropriate according to earlier reports [11] and literature antecedents [12]. The pH of this buffer, which acted as carrier, sample diluter and reagent matrix, was varied between 6·0 and 8·5; the optimum value was 7·0, although the signal increased with the pH, higher values decreased the enzyme activity through denaturation.

A 1 mmol/l coenzyme concentration was considered to be the best as it provided a baseline of 0·900 to 1·000 absorbance units. Lower concentrations shortened the activity range of the analyte that could be determined because negative absorbance values could arise in the course of the reaction and higher concentrations saturated the detector capacity.

The influence of the substrate concentration, studied in the range 0·5 to 20·0 mmol/l, was found to be optimum (highest signals) at 5·0 mmol/l.

### *Calculation of parameters*

The contribution of some variables (temperature $T$, flow-rate $q$, and injected volume $V_i$) to the mean reaction rate was calculated by plotting log $v$ versus the log of each variable; in each case, the exponent was calculated from the slope. The expression obtained was $v = K[T]^{1{\cdot}98}[q]^{-0{\cdot}26}[V_i]^{0{\cdot}92}[L_1]^{0{\cdot}59}[L_2]^{0{\cdot}20}$.

The Michaelis constant, $K_m$, was calculated at enzyme activities of 0·2 U/l. Lineweaver–Burke plots provided a value of 0·3348 $s^{-1}$ under the optimum working conditions.

### *Features of the proposed methods*

Under the optimum working conditions, MDH standards with activities between 0·02 and 2·50 U/l were injected; the calibration plot obtained were linear over the range 0·02–2·00 U/l. Table 2 lists the equations of the calibration graphs obtained for various parameters. The sensitivity (slope of the calibration graphs) can be selected as required by choosing the parameter used to run the plot, both with fixed-time measurements (sensitivity range between 0·254 and 2·744 A l/U) and reaction-rate measurements (sensitivity range between 2·853 × $10^{-3}$ and 12·293 × $10^{-3}$ $\Delta A/(\Delta t)$.l/U), with regression coefficients above 0·99 in all cases.

The reproducibility was examined by using triplicate injections of 11 different samples of MDH (activity 0·5 U/l); the relative standard deviation ranged between ±0·13 and ±0·88%.

The sampling frequency varied between 11 $h^{-1}$ ($\bar{v}'_4$) and 65 $h^{-1}$ ($A_1$ or $\bar{v}_1$).

### *Determination of malate dehydrogenase activity in blood serum*

To check its applicability the proposed method was applied to the determination of the malate dehydrogenase activity in blood sera from healthy and sick individuals obtained from a hospital. The samples were diluted to bring them within the appropriate concentration range. After the MDH activity was determined, recoveries were evaluated by adding three different amounts (0·1, 0·2, and 0·3 U/l) of standard to each sample. The results obtained from the different measurements and the recoveries are listed in table 3. The former were in the range 96–103% for the different types of measurements, which testifies the good performance of the method.

### Conclusion

Flow injection analysis has once again proved very useful for routine analyses. The small amounts of sample and reagents required for determinations and the high sampling frequency achieved make FIA method most suitable for clinical analyses. Very simple, convenient

*Table 2. Equations and features of the calibration graphs based on fixed-time (A) and reaction-rate (v) measurements.*

| Measured parameter | Equation[a] | $r^{2b}$ | Range (U/l) | R.S.D. | Sampling frequency ($h^{-1}$) |
|---|---|---|---|---|---|
| $A'_1$ | $A'_1 = 9{\cdot}2 \times 10^{-3} + 0{\cdot}254\ \lvert MDH \rvert$ | 0·9986 | 0·02–1·00 | 0·88 | 36 |
| $A_1$ | $A_1 = -1{\cdot}7 \times 10^{-4} + 0{\cdot}566\ \lvert MDH \rvert$ | 0·9994 | 0·02–1·20 | 0·64 | 65 |
| $A'_4$ | $A'_4 = 13{\cdot}6 \times 10^{-2} + 1{\cdot}091\ \lvert MDH \rvert$ | 0·9930 | 0·02–0·80 | 0·24 | 11 |
| $A_4$ | $A_4 = 13{\cdot}3 \times 10^{-2} + 1{\cdot}363\ \lvert MDH \rvert$ | 0·9994 | 0·02–0·80 | 0·19 | 14 |
| $\sum_1^4 A_i$ | $\sum_1^4 A_i = 32{\cdot}1 \times 10^{-2} + 2{\cdot}744\ \lvert MDH \rvert$ | 0·9961 | 0·02–1·00 | 0·13 | 12 |
| $\bar{v}'_1$ | $\bar{v}'_1 = 3{\cdot}18 \times 10^{-5} + 2{\cdot}853 \times 10^{-3}\ \lvert MDH \rvert$ | 0·9977 | 0·02–2·00 | 0·86 | 36 |
| $\bar{v}'_4$ | $\bar{v}'_4 = 4{\cdot}78 \times 10^{-4} + 3{\cdot}095 \times 10^{-3}\ \lvert MDH \rvert$ | 0·9902 | 0·02–1·00 | 0·21 | 11 |
| $\bar{v}'_4$ | $\bar{v}_4 = 4{\cdot}16 \times 10^{-4} + 4{\cdot}580 \times 10^{-3}\ \lvert MDH \rvert$ | 0·9993 | 0·02–0·80 | 0·19 | 12 |
| $\sum_1^4 \bar{v}'_1$ | $\sum_1^4 \bar{v}'_i = 1{\cdot}00 \times 10^{-4} + 8{\cdot}576 \times 10^{-3}\ \lvert MDH \rvert$ | 0·9961 | 0·02–1·00 | 0·13 | 11 |
| $\bar{v}_1$ | $\bar{v}_4 = 2{\cdot}36 \times 10^{-4} + 11{\cdot}770 \times 10^{-3}\ \lvert MDH \rvert$ | 0·9958 | 0·02–1·50 | 0·66 | 65 |
| $\sum_1^4 \bar{v}_i$ | $\sum_1^4 \bar{v}_i = 1{\cdot}11 \times 10^{-3} + 12{\cdot}293 \times 10^{-3}\ \lvert MDH \rvert$ | 0·9994 | 0·02–1·00 | 0·27 | 14 |

[a] Concentration of MDH in U/l.

[b] Regression coefficient.

*Table 3. Determination and recovery of MDH activity in serum samples*

| Sample | Dilution | MDH in Samples* (U/l) | Added (U/l) | Percent recovery for the different measured parameters | | | | | |
|---|---|---|---|---|---|---|---|---|---|
| | | | | $A_1$ | $A'_4$ | $\sum_1^4 A_i$ | $\bar{v}_1$ | $\bar{v}'_4$ | $\sum_1^4 \bar{v}_i$ |
| 1 | 1:100 | 0·22 ± 0·01 | 0·1 | 98 | 101 | 99 | 98 | 101 | 100 |
| | | | 0·2 | 101 | 101 | 100 | 100 | 101 | 100 |
| | | | 0·3 | 100 | 101 | 100 | 100 | 100 | 100 |
| 2 | 1:1000 | 0·48 ± 0·02 | 0·1 | 96 | 102 | 100 | 96 | 102 | 100 |
| | | | 0·2 | 101 | 101 | 100 | 100 | 101 | 100 |
| | | | 0·3 | 100 | 101 | 100 | 100 | 100 | 100 |
| 3 | 1:100 | 0·16 ± 0·02 | 0·1 | 96 | 100 | 100 | 96 | 100 | 96 |
| | | | 0·2 | 103 | 100 | 100 | 102 | 100 | 98 |
| | | | 0·3 | 100 | 100 | 100 | 100 | 100 | 99 |
| 4 | 1:100 | 0·31 ± 0·01 | 0·1 | 102 | 101 | 102 | 100 | 99 | 100 |
| | | | 0·2 | 100 | 102 | 103 | 99 | 100 | 103 |
| | | | 0·3 | 100 | 101 | 103 | 101 | 99 | 102 |
| 5 | 1:1000 | 0·23 ± 0·00 | 0·1 | 102 | 101 | 102 | 102 | 101 | 102 |
| | | | 0·2 | 100 | 100 | 99 | 100 | 100 | 99 |
| | | | 0·3 | 100 | 100 | 100 | 100 | 100 | 100 |

* Average of the different measured parameters.

instrumentation allows multidetection with a conventional single-beam spectrophotometer, thereby increasing the level of information obtained from each sample.

### Acknowledgement

The authors wish to express their gratitude to CICyT for financial support (Grant No., PA86-0146).

### References

1. Rocks, B. and Riley, C., *Clin. Chem.*, **28** (1982), 409.
2. Riley, C. and Rocks, B., *Talanta,* **31** (1984), 879.
3. Linares, P., Luque de Castro, M. D. and Valcárcel, M., *Rev. Anal. Chem.,* **VIII** (1985), 229.
4. Fernández-Romero, J. M. and Luque de Castro, M. D., *Chim. Oggi,* November (1988), 17–20.
5. Südhof, H. and Wötzel, E., *Klin. Wocheschr.,* **38** (1960), 1165.
6. Delbrück, A. and Zebe, E., *Biochem. Z.,* **331** (1959), 273–296.
7. Potter, V. R., *Journal of Biol. Chem.,* **165** (1946), 311.
8. Ríos, A., Luque de Castro, M. D. and Valcárcel, M., *Anal. Chem.,* **57** (1985), 1803.
9. Ríos, A., Luque de Castro, M. D. and Valcárcel, M., *Anal. Chim. Acta,* **179** (1986), 463.
10. Ríos, A., Lázaro, F., Luque de Castro, M. D. and Valcárcel, M., *Anal. Chim. Acta,* **199** (1987), 15.
11. Fernández-Romero, J. M. and Luque de Castro, M. D., *Fresenius Z. Anal. Chem.,* in press.
12. Almuiabed, A. M. and Townsehend, A., *Anal. Chim. Acta,* **221** (1989), 337.

